# Age-dependent changes in the anger superiority effect: Evidence from a visual search task

**DOI:** 10.3758/s13423-023-02401-3

**Published:** 2024-01-18

**Authors:** Francesco Ceccarini, Ilaria Colpizzi, Corrado Caudek

**Affiliations:** 1 Division of Science, New York University Abu Dhabi, Abu Dhabi, UAE; 2 Health Sciences Department, Università Degli Studi Di Firenze, Florence, Italy; 3 NEUROFARBA Department, Università degli Studi di Firenze, Florence, Italy

**Keywords:** Preferential processing, Angry faces, Visual search, Attention, Children, Anger superiority effect, Emotion, Threat perception

## Abstract

The perception of threatening facial expressions is a critical skill necessary for detecting the emotional states of others and responding appropriately. The anger superiority effect hypothesis suggests that individuals are better at processing and identifying angry faces compared with other nonthreatening facial expressions. In adults, the anger superiority effect is present even after controlling for the bottom-up visual saliency, and when ecologically valid stimuli are used. However, it is as yet unclear whether this effect is present in children. To fill this gap, we tested the anger superiority effect in children ages 6–14 years in a visual search task by using emotional dynamic stimuli and equating the visual salience of target and distractors. The results suggest that in childhood, the angry superiority effect consists of improved accuracy in detecting angry faces, while in adolescence, the ability to discriminate angry faces undergoes further development, enabling faster and more accurate threat detection.

A famous quote by Marcus Tullius Cicero states: “The face is a picture of the mind with the eyes as its interpreter.” The central message here is that facial expressions play a crucial role in guiding our expectations during social interactions. For instance, when we encounter someone with a happy facial expression, our expectation is typically that they are approachable, friendly, and receptive to social interaction. Conversely, when we encounter someone with an angry facial expression, our expectation is that they are unfriendly and potentially hostile.

From an evolutionary perspective, the ability to accurately detect and interpret facial expressions related to danger or threat holds significant adaptive value, because it allows us to respond appropriately to potential threats. In support of this idea, several studies have demonstrated that, in a visual search task, individuals are better able to detect an angry face among a group of different expressions than a happy face (Dixson et al., 2022; Gong & Smart, 2021; Calvo et al., 2006; Hansen & Hansen, 1988; Horstmann & Bauland, 2006; Li et al., 2022; Öhman et al., 2001; see also Rapuano et al., 2023). This effect, known as Anger Superiority Effect (ASE), highlights the unique ability of angry facial expressions to capture attention and elicit an accurate response.

The ASE has primarily been studied in adults. However, a growing body of research has turned its focus towards children, suggesting that attention biases towards threatening stimuli typically emerge early in development (e.g.,LoBue, 2009; LoBue & DeLoache, 2008; Peltola et al., 2009a, b; Reider et al., 2022).

Indeed, as early as 6–8 months of age, children demonstrate a preference for fearful and angry facial expressions (e.g., Kotsoni et al., 2001; Leppänen et al., 2018; LoBue et al., 2010; Morales et al., 2017; Peltola et al., 2018). Moreover, they exhibit slower disengagement from fearful facial configurations (e.g., Peltola et al., 2008, 2013, 2009a, b) and quicker detection of angry facial expressions compared with positive or neutral stimuli (e.g., LoBue & DeLoache, 2008; Nakagawa & Sukigara, 2012). This bias also extends to nonsocial stimuli. For instance, infants display faster attentional orientation towards snakes and spiders over frogs and caterpillars (LoBue, 2010; LoBue & DeLoache, 2008; LoBue et al., 2017).

Numerous studies have suggested that the ASE can offer valuable insights into the emotional and psychological well-being of children (e.g., Denham et al., 2002; LoBue & Pérez-Edgar, 2014; Pérez-Edgar et al., 2010; Pollak & Sinha, 2002). For instance, LoBue and Pérez-Edgar (2014) showed that children at risk for anxiety displayed an increased bias in detecting angry faces compared with low-shy comparison children. This heightened bias aligns with the findings of Pérez-Edgar et al. (2011), who reported that attention biases towards threats played a pivotal role in mediating the relationship between behaviorally inhibited temperaments and social withdrawal in children.

Conversely, maltreated children have demonstrated a heightened sensitivity to threatening stimuli, as highlighted by studies conducted by Masten et al. (2008), Briggs-Gowan et al. (2015), Swartz et al. (2011), and Pollak (2015). For instance, Pollak and Sinha (2002) found a correlation between exposure to violence or abuse and an increased likelihood of displaying the ASE, suggesting that encounters with violent events could impact their ability to recognize emotions effectively.

One particular aspect that has received meticulous attention in recent literature is the influence of low-level visual features as potential confounding factors in ASE assessments (e.g., Becker & Rheem, 2020). Converging lines of evidence suggest that the empirical results supporting the ASE are often driven by the low-level visual features of the stimulus materials, rather than by the emotion that is being portrayed (Savage et al., 2013). For instance, a number of studies (e.g., Hansen & Hansen, 1988; Horstmann & Bauland, 2006) have revealed that the ASE prevails with the Picture of Facial Affect (Ekman & Friesen, 1976), whereas the opposite effect, that is an advantage for the detection of happy faces over other facial expressions (Happiness Superiority Effect) has been often reported with the Karolinska Directed Emotional Faces (Lundqvist et al., 1998), or with the NimStim database (Tottenham et al., 2009). Horstmann et al. (2012) showed that the ASE can be confounded with the presence of diagnostic features such as the presence/absence of an open mouth with visible teeth. In particular, their research revealed that if a happy face displays teeth and an angry face does not, people are able to locate the happy face more quickly than the angry face. Conversely, if an angry face displays teeth and a happy face does not, people are able to locate the angry face more quickly than the happy face. Other studies investigated the ASE by using drawings of schematic faces (e.g., Calvo et al., 2006). However, this type of stimuli is entirely unrealistic; while they may convey negative emotions, they might not necessarily evoke a sense of threat (Becker et al., 2011). The asymmetries between angry or happy faces, typically observed when using schematic faces, may be more related to the early visual processing of line orientation than to threat detection itself (Kennett & Wallis, 2019). In line with these findings, Ceccarini and Caudek (2013) found that when bottom-up saliency is controlled, static faces do not yield an ASE. However, they did observe a reliable ASE when the same stimulus displays conveyed facial emotions through dynamic information. A possible explanation for these results may reside in the fact that, in natural conditions, emotional expressions can only be transmitted through nonrigid motions resulting from face deformations. Consequently, while static stimuli may elicit a form of processing that does not completely reflect the cognitive mechanisms naturally involved in emotion recognition (Foley et al., 2012), the use of more ecologically valid stimuli could potentially evoke stronger responses compared with static expressions. Supporting this notion, multiple studies have highlighted the significance of dynamic information for both identity and emotion recognition (LaBar et al., 2003; O’Toole et al., 2011; Thornton & Kourtzi, 2002).

While several studies have explored the impact of low-level visual features as potential confounding variables in adult ASE assessments (e.g., Becker & Rheem, 2020), this aspect has received comparatively less attention in children, especially with respect to the utilization of dynamic information. With this in mind, our current study examines the ASE in children ages 6–14 years and introduces two significant procedural modifications compared with prior research: (1) to mitigate the impact of confounding stimulus-driven factors, we designed our stimulus displays based on the guidelines set forth by Becker et al. (2011). (2) To enhance the realism and ecological validity of the stimuli, we employed dynamic facial expressions, adopting the methodology outlined by Ceccarini and Caudek (2013).

Considering that reacting promptly to potential threats offers a distinct evolutionary advantage, we anticipated that children would process angry faces more quickly and/or with greater accuracy than happy facial expressions.

## Methods and materials

### Participants

A total of 258 participants took part in the study: 85 first-grade students (36 females, *M*_age_ = 6.55 years, *SD* = 0.27), 86 fifth-grade students (41 females, *M*_age_ = 10.49 years, *SD* = 0.29), and 87 ninth-grade students (39 females, *M*_age_ = 13.70 years, *SD* = 0.26). Participants were recruited from four schools in Tuscany, Italy.

We conducted an a priori power analysis using G*Power (Version 3.1.9.7; Faul, et al., 2007) to estimate the necessary sample size. Our approach was informed by prior ASE studies (Ceccarini & Caudek, 2013; Isomura et al., 2014; May et al., 2016). We established an effect size (*f*) of 0.2 for the mean difference between neutral and angry faces in a visual search task, maintaining a significance criterion of *α* = 0.05 and power = 0.95. With this effect size, a minimum sample size of *n* = 45 was determined. Therefore, our sample size provides sufficient power to ensure meaningful and robust testing.

Two 6-year-old children were excluded from the final sample for not completing the task. This study was carried out in accordance with the Declaration of Helsinki and received approval (2015/0008103) from the local ethics committee. Informed written consent was obtained from parents, and oral consent was obtained from children (using age-adequate approaches).

### Apparatus

The experiment was controlled by MATLAB R2018a (The MathWorks, Natick, MA) using the Psychophysics Toolbox extensions (Brainard, 1997) on a PC running Windows XP. Stimuli were presented on a 19-in. video monitor operating at 75 Hz with a screen resolution of 1,280 × 1,024 pixels.

### Stimuli

**Fig. 1:**
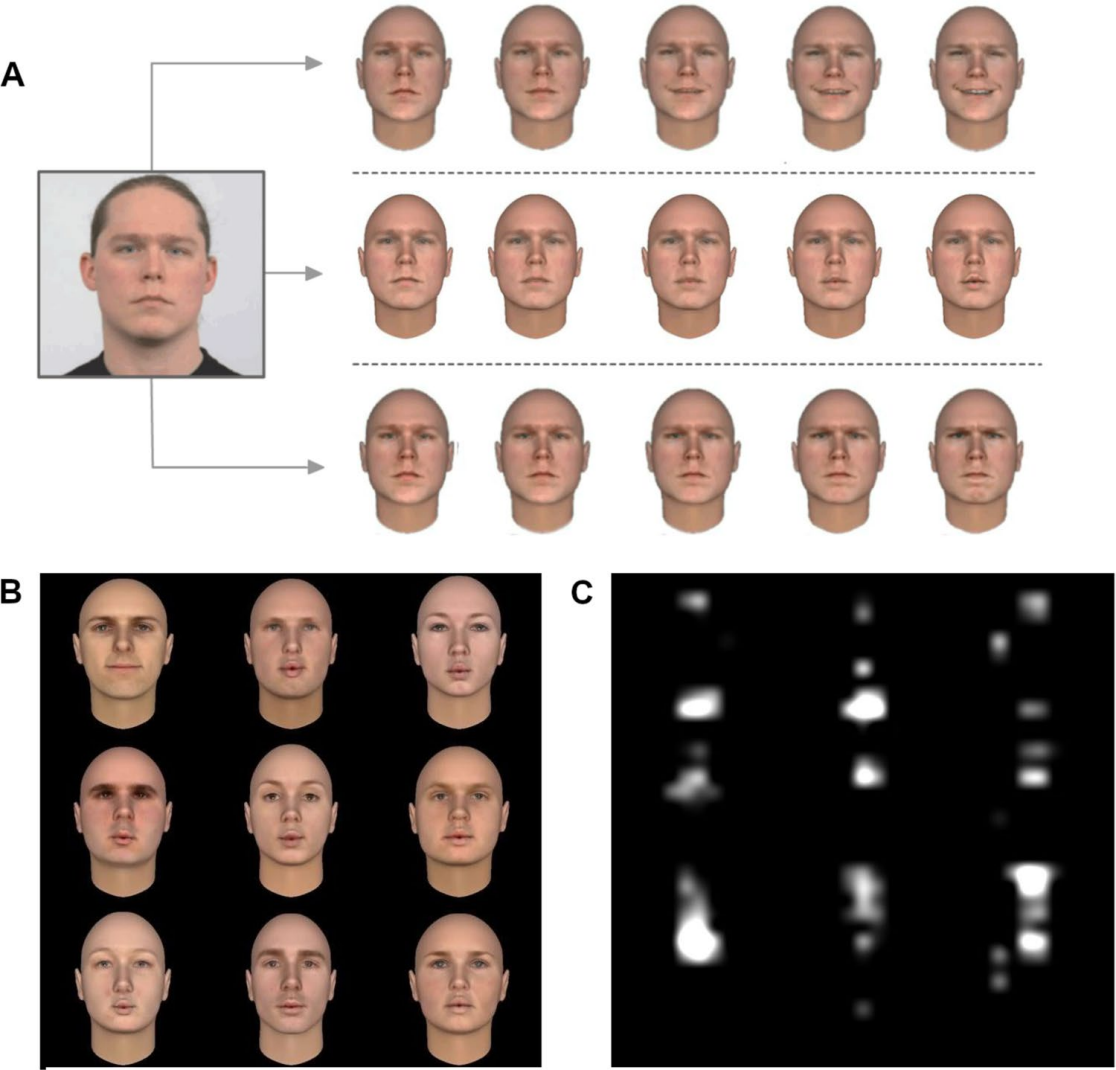
**A** Example of stimulus generation procedure. The face enclosed in the square frame was selected from the RaFD database and shows a neutral emotional expression. The image was transformed to remove hair and other distinguishing features, and then was morphed to obtain a smooth transition between the neutral expression and the full-emotion expression. **B** Example of the stimulus display. In both the simulation and the experiment, the face images were more distanced among each other (on average) and their relative positions were pseudorandomly perturbed. C Salience map of the last frame of the stimulus video

The stimuli were created by using the same procedure adopted by Ceccarini and Caudek (2013). For each emotional expression (neutral, happy, angry), we selected from the Radboud Faces Database (RaFD; Langner et al., 2010) 20 facial identities having similar ratings of intensity, clarity, and genuineness. After cropping hair and the background, by using the function PhotoFit SDK of the Facegen software, we generated a three-dimensional (3D) face model for each 200 × 200 pixels image (Fig. 1A). Each face image was then processed with 3D Studio Max, in order to equate illumination intensity and illuminant direction. These images were morphed to obtain a smooth transition between the neutral expression and the full-emotion expression (Caudek, 2013; Caudek & Monni, 2013; Ceccarini & Caudek, 2013; Lorenzino & Caudek, 2015; Lorenzino et al., 2018). The Flash CS5 software was then used to convert frame sequences into videos (Caudek et al., 2015, 2017). For generating dynamic faces with a neutral expression, we used the PhotoFit SDK function of the FaceGen software, which allows a realistic production of the spoken phoneme */W/*.

On each trial of the visual search task, participants were shown a stimulus display consisting of nine video clips of human faces showing the unfolding of an angry, happy, or neutral facial expression (Fig. 1B). Each of the nine face video sequences depicted a different facial identity. On each trial, the nine facial identities were randomly chosen from the set of 20 possible RaFD faces.

Within each stimulus display, the nine faces were displayed with a neutral expression for 300 ms, followed by the morphing transition between the neutral face and the final expressive face. The video sequence for a neutral face showed the articulation of the phoneme */W/*. The dynamic portion of the stimulus lasted for a total of 533 ms and corresponded to the presentation of the 16 images of the morph continuum, each remaining on the screen for 1/30 s. The last frame of the motion sequence remained on the screen as a static display until the participant’s response. The duration of the temporal unfolding of facial expressions of emotion is in line with previous studies (e.g., Arsalidou et al., 2011; Becker et al., 2012; Horstmann & Ansorge, 2009; Schultz & Pilz, 2009). This procedure has the advantage of allowing a precise control of the timing of the change, without sacrificing the realism of the expressive dynamics (e.g., Becker et al., 2012).

There were three conditions: eight neutral faces and an angry face, eight neutral faces and a happy face, and nine neutral faces. In half of the trials, the target was absent, and in the other half, the target (either an angry or a happy face, with equal probability) was present.

#### Bottom-up salience analysis

We selected face images having similar low-level visual features across emotional expressions. To achieve this goal, we selected a subset of the RaFD images that provided equivalent levels of bottom-up salience across happy and angry faces according to the computational model of Itti and Koch (2001). The model assumes that the allocation of visual attention is driven by stimulus salience in a bottom-up fashion and analyzes natural images by extracting low-level features such as intensity, color, and orientation at a range of spatial scales. To optimize the detection of local feature differences, these features are converted to center-surround representations; from these representations, separate “conspicuity” maps are created. The conspicuity maps are then combined to form one salience map that is supposed to guide the attentional focus. To check whether our selection procedure obtained its intended purpose, we run the following simulation. In each run of the simulations, we generated a 3 × 3 grid of different face identities, which reproduced the stimulus displays used in the experiment (Fig. 1B). One of these nine face identities was the target (with either an angry or a happy expression) and the remaining eight faces were neutral distractors faces. In each run of the simulation, the target face was positioned in one of the possible slots of the grid, with equal probability; the distractor faces (each with a different facial identity) were randomly assigned to the remaining positions of the grid. Each stimulus display was then processed with the SaliencyToolbox 2.2 (http://www.saliencytoolbox.net) for MATLAB (Walther & Koch, 2006); for the purpose of this study, the standard settings were used. An example of saliency map thus obtained is shown in Fig. 1C. From the saliency map, we measured the total activation within each of the nine regions in which the faces were located and we computed a saliency index as the ratio between the total activation of the target face and the average total activation of the distractors. This process was repeated 504 times for the angry targets and 504 times for the happy targets (by randomly varying the positions of target and distractor faces within the grid), for a total 1,008 repetitions of the simulation. The simulation results indicate that the selected angry and happy faces showed very similar levels of bottom-up salience according to Itti and Koch’s metrics: saliency index difference, 0.01 ± 0.02 *SE*; 95% credibility interval, [− 0.03 to 0.05].

#### Amount of image motion

For the twenty selected RaFD face identities, we estimated the amount of image motion generated by the temporal unfolding of a happy or angry facial expression, or for the articulation of the phoneme */W/*. The amount of image motion was computed as indicated by Ceccarini and Caudek (2013). We divided the squared I (*x*, *y*, *t*).

greyscale images at the time t_1_ (first frame of the video sequence) and I_2_ (last frame of the video sequence) into a grid of smaller 20 × 20 blocks. We summed the values within each block to obtain the reduced images I_r_ (t_1_) and I_r_ (t_2_). Image motion was then estimated as the sum of all elements of the absolute difference of I_r_ (t_1_) and I_r_ (t_2_).

Results indicated that, on average, the three facial expressions produced similar amounts of image motion—happy: *mean*, 0.91 ± 0.193 *SE*; angry: *mean*, 0.98, ± 0.231 *SE*; neutral: *mean*, 0.88 ± 0.196 *SE*; *F*(2, 3) = 1.22, *p* = 0.3030.

#### Intensity of emotional expressiveness

The emotional intensity of the 20 selected RaFD faces was evaluated by 94 undergraduate students. Static images of the happy and angry faces were presented for 15 s in random order. Participants were asked to rate the emotional intensity of each face on a scale ranging from 1 (*very low intensity*) to 4 (*very high intensity*). We found no evidence that perceived emotional intensity varied across the two facial expressions (happy: mean, 2.98 ± 0.025 *SE*; sad: *mean*, 3.01 ± 0.021 *SE*; *z* = 0.16, *p* = .8729).

### Procedure

Participants were tested in individual sessions in a quiet room at their school. The experimenter introduced the study as a game. Prior to test trials, participants completed nine practice trials (three with a happy target face, three with an angry target face, and six in the distractor-only condition). Responses to practice trials were excluded from further analyses. If the experimenter judged that the participant understood the task, and if the children also gave their oral consent to continue, the experiment started. Each trial of the visual search task began with the presentation of a central fixation point for 1,000 ms, which was followed by a display containing nine dynamic faces. Participants were asked to indicate with a key-press whether all faces showed the same expression or whether one face showed an expression differing from the others. They were instructed to perform the task as quickly and accurately as possible. Participants received no feedback for correct or incorrect responses. Participants completed the study in a single session lasting approximately 25 minutes, including the breaks between blocks of trials.

### Data analysis

Outliers were identified as data points above the third quartile or below the first quartile of 1.5 interquartile ranges, and these were excluded from analysis (Tukey, 1977), resulting in the removal of 1.5% of total trials. Reaction time (RT) distributions, measured in seconds, were analyzed using the ex-Gaussian distributional model (Balota & Yap, 2011). In the ex-Gaussian model, RT distribution is represented as the convolution of two random variables: one normally distributed with mean *µ* and standard deviation *σ*, and the other exponential with mean and variance equal to *τ*.

The ex-Gaussian mean equals *µ* + *τ*, and its variance is *σ*^2^ + *τ* ^2^ (Luce, 1986). Bayesian hierarchical estimation was employed due to its ability to better estimate ex-Gaussian distribution parameters with limited observations compared with maximum likelihood estimation (Rouder et al., 2005).

To estimate participants’ detection ability and response bias, we employed a Bayesian hierarchical extension of the Signal Detection Theory model (SDT; Green & Swets, 1966), which offers advantages over conventional methods (Lee & Wagenmakers, 2014). The posterior distribution of the hierarchical SDT model was estimated via MCMC using the Stan package (Carpenter et al., 2016) within the R statistical language (R Core Team, 2016). Samples were generated from two independent chains, each comprising 50,000 iterations, with 5,000 warm-up iterations. Convergence was assessed using the $\hat{R}$ statistic (Gelman & Rubin, 1992).

The 95% Bayesian credible intervals (indicating the range within which we can be 95% certain that a parameter’s true value lies given the observed data) were used for statistical inference. In contrast, a frequentist confidence interval reveals that, if the statistical procedure were applied repeatedly to various hypothetical datasets, the confidence interval would encompass the true parameter in 95% of cases. Therefore, the frequentist CI provides insight into the properties of the statistical procedure being employed, but not the parameter’s uncertainty. Conditions were deemed different if their credible intervals did not overlap. We computed the standardized effect size using the *δ*_*t*_ index (Nalborczyk et al., 2019).

## Results

### Hierarchical ex-Gaussian fit

**Fig. 2:**
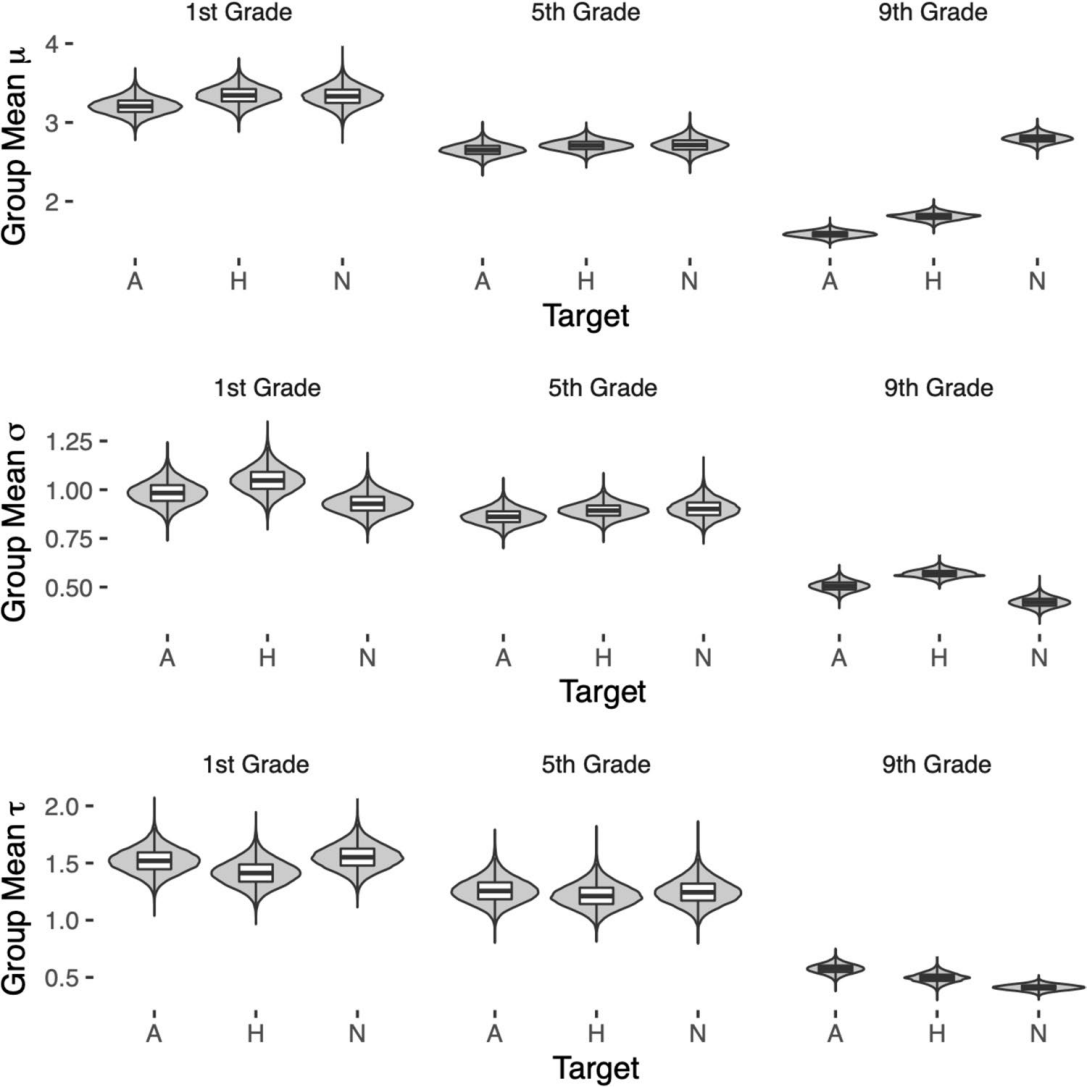
Hierarchical Bayesian estimates of the parameters µ, σ, and τ (all in seconds) of an ex-Gaussian distribution fitted to the data of each experimental condition for the three age groups

Figure 2 shows the posterior distributions of the hierarchical Bayesian estimates of the parameters *µ*, *σ*, and *τ* (all in seconds), for each age group and experimental condition. In terms of RTs, first-grade and fifth-grade students did not show any evidence of prioritization for angry faces (see Table 1). Conversely, ninth-grade students detected angry targets quicker than happy ones.

**Table 1:** Posterior medians and the 95% credible intervals of hierarchical Bayesian estimates of the parameters µ, σ, and τ of the ex-Gaussian distribution fitted to the data (in seconds) of each experimental condition for the three age groups: A: angry face target; H: happy face target; N: target absent

		I Grade	
	*µ*	*σ*	*τ*
A	3.21 (3.00, 3.42)	0.98 (0.87, 1.10)	1.52 (1.31, 1.74)
H	3.35 (3.12, 3.58)	1.05 (0.92, 1.18)	1.41 (1.20, 1.64)
N	3.33 (3.09, 3.58)	0.93 (0.83, 1.04)	1.55 (1.34, 1.77)
		V Grade	
	*µ*	*σ*	*τ*
A	2.65 (2.51, 2.81)	0.86 (0.78, 0.95)	1.26 (1.05, 1.49)
H	2.71 (2.57, 2.85)	0.89 (0.82, 0.97)	1.21 (1.01, 1.43)
N	2.72 (2.55, 2.89)	0.90 (0.81, 1.00)	1.25 (1.04, 1.47)
		IX Grade	
	*µ*	*σ*	*τ*
A	1.58 (1.50, 1.67)	0.51 (0.45, 0.56)	0.57 (0.49, 0.66)
H	1.81 (1.71, 1.90)	0.57 (0.53, 0.61)	0.50 (0.41, 0.58)
N	2.80 (2.68, 2.91)	0.42 (0.37, 0.47)	0.41 (0.36, 0.46)

### Hierarchical SDT analysis

Table 2 shows the *d′* posterior estimates as a function of target type and age groups. Results shows a detection advantage, in all three age groups, for angry target faces over happy target faces. The results are clear enough. But, if one desires to combine a Bayesian “estimation” approach with the frequentist 95% window size for decision making, then it can be noted that the 95% credible intervals of angry and happy target faces do not overlap for first-grade students (the crucial age group), effect size = 0.535, but they do overlap for the two older age groups. To obtain a more precise posterior estimate with a larger sample, we fitted the model again after collapsing the two older age groups. By so doing, we found the following. Angry face targets: *µ*_d_ = 3.02; 95% credible interval, 2.85 to 3.19; happy face targets: *µ*_d_ = 2.64; 95% credible interval, 2.49 to 2.80; effect size = 0.40. We thus conclude that, in terms of detection performance, the ASE is present in both younger and older children. The effect size is in the small-medium range of Cohen’s (1988) effect size benchmarks.

**Table 2:** Posterior medians and 95% credible intervals of hierarchical SDT model for the three-age group

	I Grade	Sensitivity *µ*_d_	IX Grade
		V Grade	
A	2.87 (2.60, 3.15)	2.91 (2.65, 3.19)	3.10 (2.90, 3.31)
H	2.32 (2.29, 2.58)	2.51 (2.26, 2.75)	2.74 (2.55, 2.94)

### Summary

Hierarchical ex-Gaussian fit indicates angry faces are detected quicker than happy faces only in the older age group. The first-grade and fifth-grade students showed no prioritization for threatening faces. Conversely, detection performance was similar across all age groups. Children were always better at detecting an angry target than a happy target.

## General discussion

The primary objective of this study was to explore the ASE across various age groups, encompassing first-grade, fifth-grade, and ninth-grade students. For this purpose, we examined the ASE in young children using dynamic ecologically valid stimuli, and controlling for low-level perceptual confounds. Our results indicate that, during childhood, the ASE manifests as enhanced accuracy in detecting angry faces, while in adolescence, the ASE undergoes additional refinement, leading to both quicker and more precise threat detection. The fact the younger children detect more accurately an angry than a happy target is in line with the previous literature holding that threatening stimuli benefit of an enhanced perceptual encoding from an early age (e.g., LoBue & DeLoache, 2008). However, we found that a quicker detection of angry faces was present only in the older age group (ninth-grade students). A possible explanation for this result may reside in the substantial neurobiological changes occurring during the transition from childhood into adolescence.

Specifically, the development of facial expression recognition constitutes a multifaceted process that involves the maturation of various brain regions, encompassing the occipito-temporal areas responsible for analyzing the holistic perceptual layout of visual facial features. Additionally, an “emotional network,” including the anterior temporal cortex, precuneus, anterior paracingulate cortex, inferior frontal gyrus, amygdala, insula, and the reward system (Duchaine & Yovel, 2015; Gobbini & Haxby, 2007; Haxby et al., 2001; Maffei & Sessa, 2021a, b), contributes to the analysis of facial expressions. During development, this emotional network undergoes noteworthy structural changes (Kanwisher et al., 1997; Thomas et al., 2001) that impact the capacity to process and differentiate facial expressions, particularly those with threatening features (Herba et al., 2006; Herba & Phillips, 2004; Montirosso et al., 2010; Vicari et al., 2000).

Supporting this notion, earlier studies have revealed that children ages 4–8 years exhibit reduced accuracy in recognizing and distinguishing facial expressions in contrast to adolescents and adults (Herba & Phillips, 2004). Only during late childhood do children begin to approach levels of accuracy akin to those of adults in recognizing and discriminating facial expressions (Herba & Phillips, 2004; Herba et al., 2006; Montirosso et al., 2010; Vicari et al., 2000). In light of these findings, the refinement of the ASE during adolescence could be interpreted as an epiphenomenon of the ongoing maturation of the emotional network and the cortical areas related to facial expression discrimination and recognition.

It remains to be explained why some studies have reported a quicker detection for angry faces (e.g., May et al., 2016), while others have reported a faster detection for happy faces (e.g., Leppänen & Hietanen, 2004; Zsido et al., 2021) in children.

One possible explanation for this discrepancy could be linked to the stimuli utilized in prior research. In general, except for a few exceptions (e.g., Ceccarini & Caudek, 2013), investigations into the ASE in children have primarily employed artificial schematic stimuli or static images depicting facial expressions, without adequately considering low-level factors. This might have impacted the visual prominence of distinct facial expressions, potentially introducing a bias that could lead to a faster detection of angry or happy targets. Detecting emotionally charged stimuli is a complex process influenced by both (1) bottom-up factors enhancing visual distinctiveness and (2) top-down attention towards emotional stimuli like angry or happy faces. Demonstrating a “superiority effect” based on emotional content requires showing this advantage persists even when minimizing low-level features. This precisely defines our objective in the present study, and our results indicate that an “anger superiority effect” consistently emerges, even in children.

A distinguishing aspect of our study is the use of dynamic stimuli. Prior research has demonstrated that dynamic facial expressions facilitate improved emotion recognition in comparison to static facial expressions (Frijda, 1953; Harwood et al., 1999; Kozel & Gitter, 1968). Dynamic expressions are rated as more intense than static emotional faces (Biele & Grabowska, 2006) and enable more precise identification. The relevance of dynamic information becomes particularly evident in situations with limited available physical data (Ambadar et al., 2005; Bould et al., 2008) or compromised data (Kätsyri & Sams, 2008; Wallraven et al., 2008). Furthermore, dynamic information is beneficial in clinical contexts. For example, individuals with intellectual disabilities (Harwood et al., 1999), pervasive developmental disorder (Uono et al., 2010), and autism (Back et al., 2007; Gepner et al., 2001) gain advantages from dynamic stimuli, resulting in enhanced recognition of facial expressions. Interestingly, earlier studies have highlighted that perceiving and recognizing static or dynamic facial expressions involve distinct neural pathways (Kilts et al., 2003). In static facial expressions, the perception of anger activates a cortical network encompassing motor, prefrontal, and parietal regions. Conversely, the perception of anger in dynamic facial expressions is linked to heightened right-lateralized activity in the medial, superior, middle, and inferior frontal cortex, as well as the cerebellum. Electromyography studies further support this dissociation, revealing that dynamic expressions tend to evoke more pronounced facial mimic responses and are associated with elevated physiological activation levels (Alves, 2013). Considering the above, utilizing dynamic stimuli facilitates the implementation of a more genuine and ecologically valid task, thereby offering an enhanced opportunity to investigate the ASE (Ceccarini & Caudek, 2013).

## Conclusion

The present study provides compelling evidence for the existence of the ASE in children. Specifically, our results confirm that threatening stimuli benefit from an enhanced perceptual encoding from an early age (e.g., LoBue, 2009; LoBue & DeLoache, 2008; Peltola et al., 2009a, b; Reider et al., 2022), substantiating the notion that humans possess a dedicated mechanism for detecting potential threats and directing attentional resources toward them (Öhman & Mineka, 2001). Within this context, the ASE can be interpreted as an adaptive mechanism evolved to prioritize the identification of a potent social warning signal, such as angry faces (Hansen & Hansen, 1988; Horstmann & Bauland, 2006; Öhman et al., 2001).

However, it’s important to acknowledge that this study is not without limitations. Firstly, children were required to discriminate facial expressions with emotional intensity validated in a sample of adult participants. Thus, the emotional intensity of our stimuli might not precisely align with children’s developmental ability to discern facial expressions. Secondly, while our cross-sectional design offers a snapshot of the ASE across different age groups, a longitudinal approach would yield a more comprehensive understanding of the ASE’s developmental trajectory. Lastly, despite employing a validated method to control the bottom-up saliency of our stimuli, other low-level factors could still have impacted our results. Hence, further research is essential to validate and generalize the present findings.

## Data Availability

All relevant materials, data and R scripts are available (https://github.com/ccaudek/ase_children).
